# Pathogenetic detection, retrospective and pathogenicity analysis of a fatal case of *Vibrio vulnificus* in Shenzhen, China

**DOI:** 10.1186/s13099-023-00580-x

**Published:** 2023-11-25

**Authors:** Shiqin Xu, Jinsong Wu, Ying Jin, Liyin Ji, Xuan Zou, Qinghua Hu, Tiejian Feng, Shuang Wu, Yixiang Jiang, Qiongcheng Chen, Huiqun Lu, Shuxiang Qiu, Huaisheng Chen, Min Jiang, Rui Cai, Yaqun Qiu, Xiaolu Shi

**Affiliations:** 1 School of Public Health, Shanxi Medical University, 030001 Taiyuan, China; 2 Shenzhen Center for Disease Control and Prevention, Shenzhen, 518055 China; 3 Shenzhen Research Center for Communicable Disease Control and Prevention, Chinese Academy of Medical Sciences, Shenzhen, China; 4 Shenzhen People’s Hospital, Shenzhen, 518020 China; 5 Shenzhen Bao’an District Songgang People’s Hospital, Shenzhen, 518105 China; 6 School of Public Health, University of South China, 421001 Hengyang, China

**Keywords:** *Vibrio vulnificus*, Food Poisoning, Antibiotic susceptibility, Virulence testing, Genome sequencing

## Abstract

We report a 36-year-old male patient died of *V. vulnificus*-induced septicaemia and multiple organ failure syndrome after oyster consumption at a restaurant. We isolated and identified *V. vulnificus* vv16015 from the patient’s blood sample and antibiotic susceptibility tests indicated sensitivity to all 21 antibiotics. Oyster samples were subsequently collected from the restaurant’s supplier and three strains of *V. vulnificus* were isolated. Whole genome sequencing and analysis revealed vv16015 to be distantly related to these strains and confirmed that *V. vulnificus* contamination was present in the seafood of the restaurant and supplier. Using a *Galleria mellonella* larvae infection model, the virulence of vv16015 was determined to be higher than that of comparison strains isolated from a surviving patient (vv15018) and an oyster (vv220015). The human and environment distribution of *V. vulnificus* in Shenzhen is sporadic and heterogeneous, and vv16015 is highly virulent compared to other strains.

**Supplementary Information:**

The online version contains supplementary material available at 10.1186/s13099-023-00580-x.

## Introduction

*Vibrio vulnificus* is a potentially deadly opportunistic and emergent human pathogen [1]. Infection with *V. vulnificus* typically occurs through broken skin-contact with seawater or seafood consumption [1], though it has been reported from a bee sting [2]. *V. vulnificus* infection usually causes mild gastroenteritis, severe wound infection and sepsis [3]. The mean incubation period for *V. vulnificus* infection is 16 h for wound infection and 26 h for sepsis, with mortality rates of up to 50% and 25%, respectively [4]. Although *V. vulnificus* infections are rare, it disproportionately affects men, and patients with underlying health conditions, including liver disease (cirrhosis or hepatitis), diabetes mellitus and immune dysfunction, are at highest risk of mortality [5]. *V. vulnificus* has the highest mortality rate of all foodborne pathogens, and accounts for 94% of *Vibrio* spp.-related deaths, with the exception of *V. cholerae* [6].

While cases of *V. vulnificus* infection are likely underreported due to frequent mild symptoms, the majority of reports are from the United States of America (USA), Japan, South Korea, and China [7]. In China, *V. vulnificus* infection cases are predominantly reported in the south-eastern coastal region, including the regions of Guangdong, Taiwan, Zhejiang, and Fujian, corresponding with warm seawater conditions that are optimal for the pathogen [7]. In the USA, between 1992 and 2007, there was a mortality rate of 51.6% among 459 *V. vulnificus* infections [8]. A higher mortality rate was reported in Japan at 63.2% for 185 patients between 1975 and 2005 [9] and 34 cases of infection due to seafood consumption in South Korea (2000–2011) had a mortality rate of 47.1% [10]. In China, eleven *V. vulnificus*-infected patients of 33 died (33.3%) in Guangdong Province between 2010 and 2020 [9], and similar mortality rates were reported in Taiwan (24%) [11] and Zhejiang (19.1%) [12]. Shenzhen is a coastal city in the Guangdong Province, with numerous *Vibrio* spp. present in that water of local beaches [13], however, no deaths resulting from *V. vulnificus* infection have previously been reported.

We report a case of foodborne *V. vulnificus* infection from oyster consumption in Shenzhen, Guangdong Province, China, which led to a rapid deterioration of the patient’s condition and death on day two post-admission. We isolated *V. vulnificus* strains from the patient’s blood and oyster samples, tested antimicrobial susceptibility and modeled the virulence. The study provides insight into the variable virulence of the causative strain local seafood *V. vulnificus* contamination and variable virulence, which may be cause of concern.

## Materials and methods

### Bacterial isolation and identification

*V. vulnificus* was isolated from the patient’s blood sample using a blood culture on modified cellobiose-polymyxin B-colistin (mCPC) agar medium and purified on blood plate agar medium. *V. vulnificus* was identified using Matrix-Assisted Laser Desorption/Ionization**-**Time Of Flight Mass Spectrometry (MALDI-TOF MS) and COMPACT VITEK2 and named vv16015.

### Ethics statement

All animal experiments were approved by the Clinical and Research Ethics Committee of Shenzhen Centre for Diease Control and Prevention.

### Antimicrobial susceptibility testing (AST)

Susceptibility of vv16015 to 21 antibiotics studied using the VITEK 2 AST-GN09 antimicrobial susceptibility board: amikacin, ampicillin, ampicillin/sulbactam, aztreonam, cefazolin, cefepime, cefotetan, ceftazidime, ceftriaxone, cefuroxime, ciprofloxacin, gentamicin, imipenem, levofloxacin, meropenem, nitrofurantoin, piperacillin, piperacillin/tazobactam, tobramycin, trimethoprim/sulfamethox, and tetracycline. The results were automatically interpreted by the VITEK 2-COMPACT fully automated microbiological analyser, using *E. coli* ATCC25922 as the quality control strain, according to the recommendations of the Clinical and Laboratory Standards Institute document M100-S30 [14].

### Genomic DNA extraction and bioinformatics analysis

Genomic DNA was extracted using the QIAamp DNA Mini Kit and whole genome sequencing was performed by BGI (Shenzhen, China). Raw data were filtered using Trimmomatic v0.39 [15], SPAdes v3.9.1 [16] was used to perform *de-novo* genome assembly. Kraken2 (https://ccb.jhu.edu/software/kraken2/) was used to identify *V. vulnificus*, Prokka 1.14.6 [17] was used for gene annotation. The vv16015 carries virulence genes identified through the VFDB online database (http://www.mgc.ac.cn/VFs/) and resistance genes identified through CARD online database (https://card.mcmaster.ca/).

From the National Center for Biotechnology Information (NCBI), we downloaded the genome sequences of 133 *V. vulnificus* strains isolated from eight countries between 1993 and 2022. To show the evolutionary relationships between vv16015 and the 133 other *V. vulnificus* strains, a maximum-likelihood phylogeny was constructed, with CMCP6 as the reference genome as it was considered to be the most complete and accurate of the published *V. vulnificus* clinical strain genomes. Snippy Pipeline v4.6.0 was used to identify core genomic single nucleotide polymorphisms (core-SNPs), then Maximum-likelihood phylogeny was constructed using fasttree and visualised and annotated in iTOL.

### *Galleria mellonella* virulence assay

The *Galleria mellonella* larvae model is widely used to study the pathogenic potential of *Vibrio* as it is easily survivable, simple, inexpensive and more ethically acceptable than mammalian infection models [18]. We established a *G. mellonella* larvae model to study the virulence of *V. vulnificus* vv16015, vv15018 and vv220015 were selected for experimental comparison and sourced from the Shenzhen Centre for Disease Control and Prevention strain bank. Vv15018 was isolated from a clinical patient with no specific disease history and discharged from hospital in good condition after an acute gastroenteritis caused by a poor diet. Vv22015 was isolated from an oysters and represents an environmental strain that was used as the experimental control because its virulence genes are approximately the same as those of vv16015 and vv15018.

Sixth instar *G. mellonella* larvae were 2-3 cm long with a mean weight of 0.33 g (Tianjin Huiyude Biotechnology Company Limited) and selected for vigour and absence of black spots. There were seven groups each with ten larvae. One group remained untreated controls (group C1) and six groups were injected with 10µL of control or bacterial suspension on the last right-side pair of hind feet. Group C2 received 0.9% sterile saline, T1 received 1 × 10^4^ CFU/mL, T2 1 × 10^5^ CFU/mL, T3 1 × 10^6^ CFU/mL, T4 1 × 10^7^ CFU/mL and T5 1 × 10^8^ CFU/mL. After injection *G. mellonella* larvae were placed in separate sterile disposable petri dishes and incubated in a constant temperature incubator at 37 °C for 48 h and survival curves were plotted for *G. mellonella* larvae. Survivorship was recorded every 6 h.

### Statistical analysis

All statistical analyses were performed using GraphPad Prism 9.5.0 software. Larval survival were plotted by the Kaplan-Meier method. Log-rank (Mantel-Cox) test was used to compare the significance of differences in larval survival rates between groups. P < 0.05 was considered to indicate significant differences between groups.

## Case report and results

A 36-year-old male patient with a history of liver cirrhosis and no traceable traumatic history consumed chilled oysters at a seafood restaurant in Shenzhen, China, on August 28, 2016. He felt unwell at approximately 16:00 on August 29th, 2016, and reported dizziness and a temperature of 39 °C. Symptoms progressed to include diarrhoea and abdominal pain at 19:00 on the 29th, and calf muscle pain at 4:00 on the 30th. He arrived at hospital at 5:00 on the 30th. We took treatment with ulinastatin for anti-shock, meropenem for anti-infection, continuous ventilator-assisted breathing, adenosylmethionine butanedisulfonate for hepatic protection, esomeprazole for acid suppression and gastric protection, human immunoglobulin for immune enhancement, plasma transfusion for improved oxygenation, bedside haemofiltration, and other treatments administered. Death was recorded on August 31, 2016 at 16:41 from *V. vulnificus*-induced sepsis combined with multiple organ failure syndrome, rhabdomyolysis syndrome, active chronic viral hepatitis B, and post-hepatitis B cirrhosis. The person who had eaten at the restaurant with the patient reported diarrhoea symptoms on August 29-30th, though an anal swab was negative for pathogenic bacteria.

Antimicrobial susceptibility testing (AST) demonstrated susceptibility of vv16015 to all 21 antibiotics.We collected 13 oyster samples from the restaurant that the patient has eaten at and were unable to isolate *V. vulnificus*. 20 oyster samples were collected from the restaurant’s supplier and from which we isolated three strains of *V. vulnificus*: vv35, vv36, and vv37, which were sent with vv16015 to BGI (Shenzhen, China) for whole genome sequencing.

**Fig. 1:**
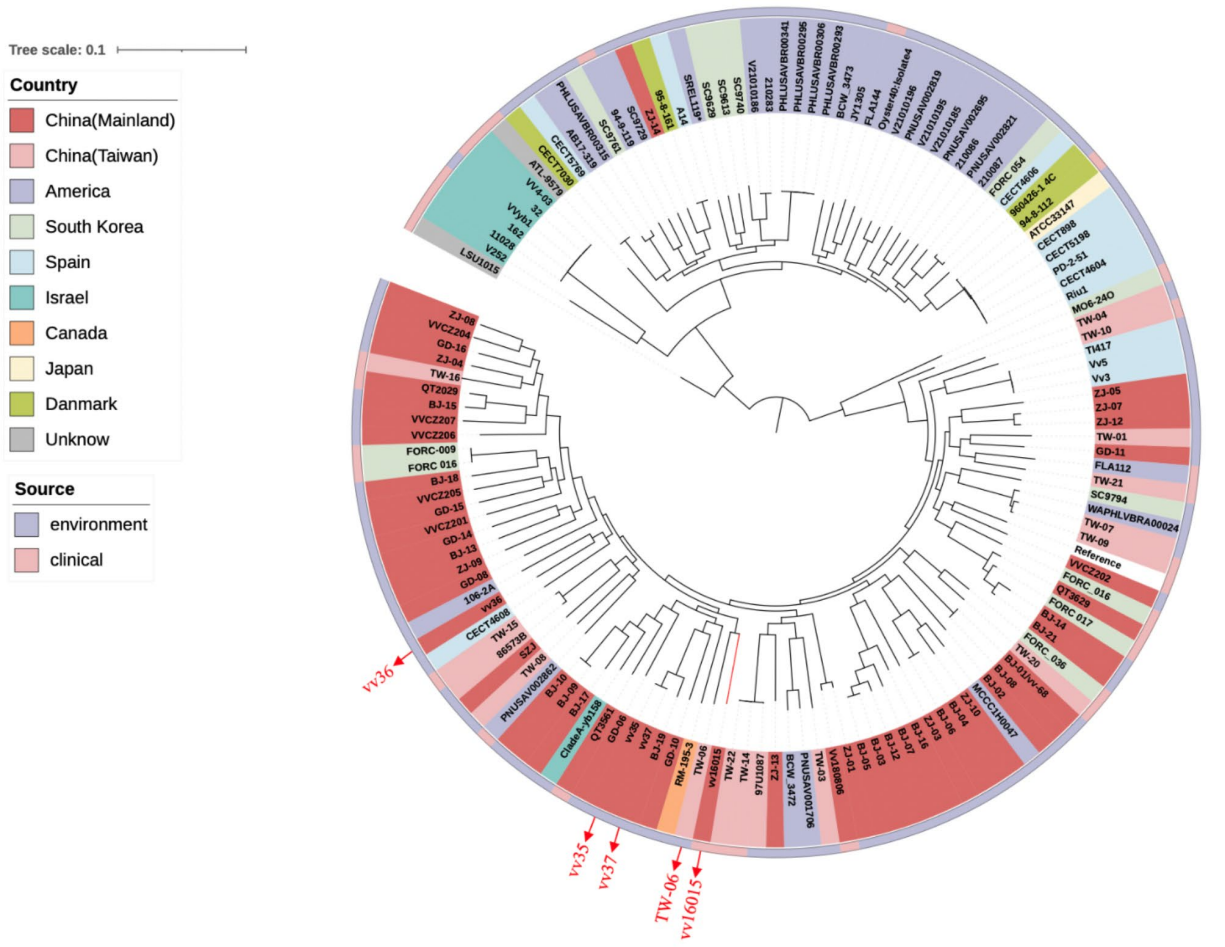
Maximum-likelihood phylogeny of vv16015 with other *V. vulnificus.* (vv16015 is marked with a red branch)

The genome size of vv16015 is 4.9 Mb, with a GC content of 46%, including 4306 coding sequence (CDS). Vv16015 has a total of 126 virulence genes, approximately equivalent to vv35, vv36 and vv37, and 1 resistance gene, *tet (34)*. Pathogenesis-associated genes including *vvhA*, *rtx* gene cluster, and virulence genes associated with iron uptake in vv16015 suggests that the high pathogenicity of this strain is associated with a mixture of virulence factors. Maximum-likelihood phylogeny showed that vv16015 is distantly related to vv35, vv36, and vv37 in different branches (Fig. 1), in contrast, is specifically assigned to branch TW-06, and therefore most similar to *V. vulnificus* isolated from oysters in Taiwan, China.

**Fig. 2:**
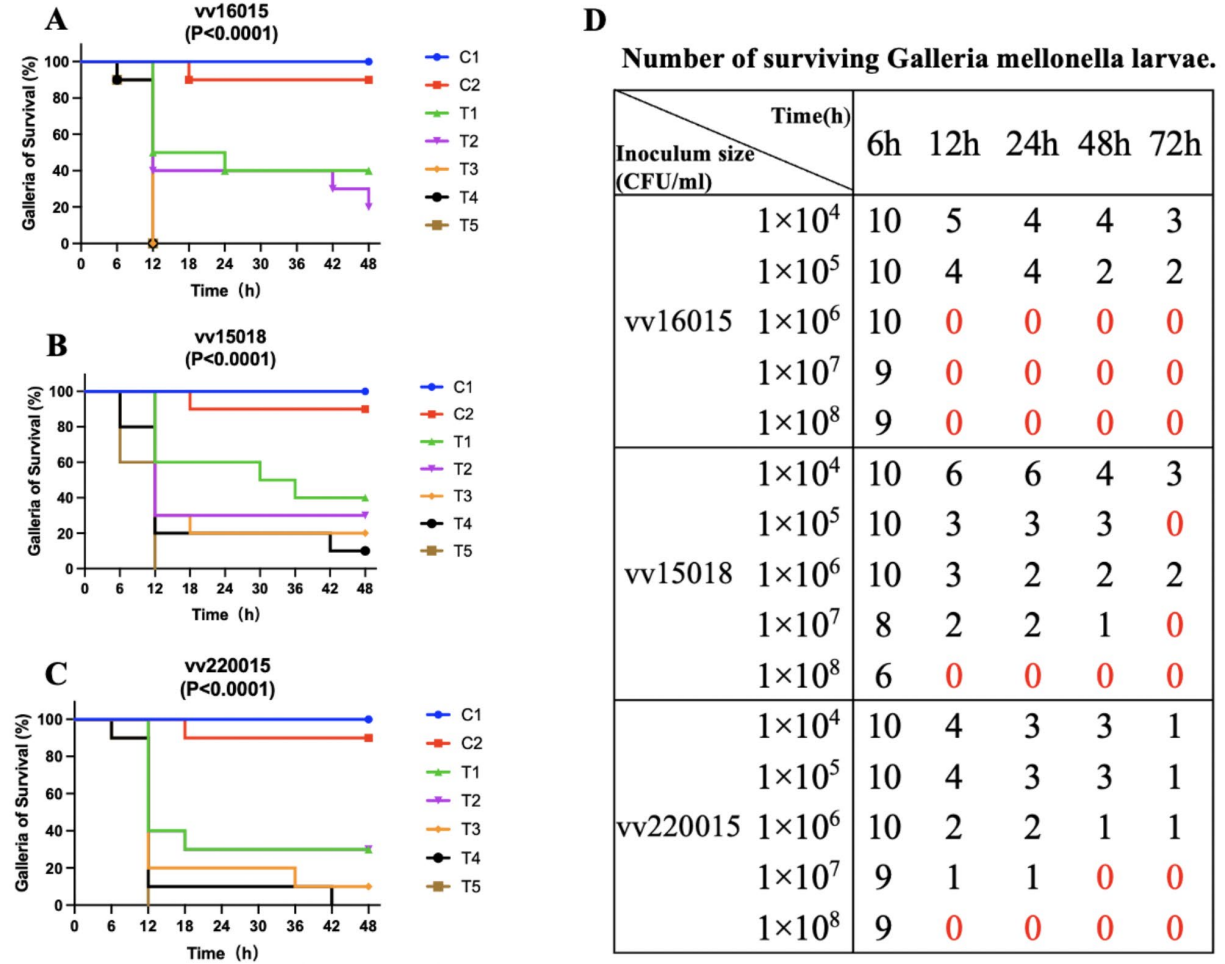
Kaplan-Meier survival curves of *G. mellonella* larvae infected with *V. vulnificus* strains at different inoculum sizes. (A) vv16015, (B) vv15018, and (C) vv220015. C1 = untreated control; C2 = saline control; T1 = 1 × 10^4^ CFU/mL; T2 = 1 × 10^5^ CFU/mL; T3 = 1 × 10^6^ CFU/mL; T4 = 1 × 10^7^ CFU/mL; T5 = 1 × 10^8^ CFU/mL. (D) Number of surviving *G. mellonella* larvae after inoculation with different inoculum sizes

The survival outcomes of *G. mellonella* larvae were dependent on both inoculum size and *V. vulnificus* strain (Fig. 2). A 100% mortality rate was observed in strain vv16015 at 12 h with an inoculum size of 1 × 10^6^ CFU/mL, as compared with 30% and 20% mortality rates of vv15018 and vv220015, respectively. Vv15018 reached 100% mortality at 1 × 10^7^ CFU/mL for 72 h and vv220015 at 1 × 10^7^ CFU/mL for 48 h (Fig. 2). The larvae of the untreated (C1) and saline control (C2) groups had 0% cumulative mortality at 12 h. Meanwhile, vv16015, vv15018 and vv220015 showed significant differences (P < 0.001) in the survival rate of *G. mellonella* larvae after inoculation with different inoculum sizes (Fig. 2), indicating that the virulence of these strains on larvae was dose-dependent, and the higher the inoculum size, the more and faster the larvae died. Furthermore, there was a significant difference between the comparator strains vv15018 and vv220015, and the patient strain vv16015 isolated in this study, which proved to be the most virulent (vv15018 vs. vv16015, P = 0.011; vv220015 vs. vv16015, P = 0.019).

## Discussion

Infections caused *Vibrio vulnificus* are rare but increasing, and the mortality rate among vulnerable patients is high [4], suggesting an increasing cause for concern. We report the first patient death from *V. vulnificus* infection in Shenzhen, China, which was likely complicated by a history of cirrhosis.

The *V. vulnificus* infection strain isolated from the patient (vv16015) was phylogenetically distinct from the three strains (vv35, vv36, vv37) isolated from oysters obtained from local restaurant suppliers in Shenzhen, and most similar to strain TW-06 isolated from oysters from Taiwan, Chinad (Fig. 1). This suggests that the population composition of *V. vulnificus* is genetically diverse and heterogeneous in the areas from which seafood is imported Shenzhen, consistent with previous findings of contaminated seafood and *Vibrio* spp. isolated from Chinese waters [19]. Furthermore, the discovery of several *V. vulnificus* strains within the local seafood supply chain alerted us to potentially dangerous contamination.

The pathogenesis of *V. vulnificus* is not yet fully understood, although several virulence factors have been independently identified, whether these virulence factors are key factors in *V. vulnificus* disease pathogenesis is largely unknown [5]. The virulence genes of vv16015 were broadly consistent with other *V. vulnificus* virulence genes. While, based on previous studies [6], vv16015 was predicted to have resistance gene *tet (34)*, which encodes an oxytetracycline resistance phosphoribosyltransferase that mediates tetracycline resistance, AST demonstrated tetracycline sensitivity. Such sensitivity to this antibiotic which may be due to acetylation-mediated down-regulation of the gene *tet (34)* [6]. Although the AST results showing the sensitivity of vv16015 to all 21 antibiotics suggest that this patient may have missed the best early treatment time. It also reminds us that timely treatment with broad-spectrum antibiotics may have increased the chances of patient survival. The higher virulence of vv16015 than comparative strains of *V. vulnificus* was demonstrated using the *Galleria mellonella* larvae model [20],and may explain the patient outcome.

## Conclusions

This is the first reported case of death related to *V. vulnificus* infection in Shenzhen, China. Although *V. vulnificus* infections are rare, due to the high mortality rate in patients with pre-existing conditions and unclear pathogenesis, we recommend systematic surveillance of seafood for *V. vulnificus* contamination. Furthermore, we suggest that clinicians provide health education to patients with underlying illnesses that place them at higher risk, recommending avoidance raw or undercooked seafood and contact with seawater with broken skin.

### Electronic supplementary material

Below is the link to the electronic supplementary material.

Supplementary Material 1

## Data Availability

Sequence data are available in the NCBI BioProject database under the accession number, PRJNA960661. All other data are available in the manuscript.
