# Supplementary material for: Pathogenetic detection, retrospective and pathogenicity analysis of a fatal case of Vibrio vulnificus in Shenzhen, China
Source: Gut Pathog. 2023 Nov 25;15:57. doi: 10.1186/s13099-023-00580-x (PMC10675978; doi:10.1186/s13099-023-00580-x)
Supplement: Supplementary file 1 — Supplementary Material 1 [file 13099_2023_580_MOESM1_ESM.pdf]

Ethics Committee of Shenzhen Centre for Disease Control and Prevention

Ethical Review Approval

Approval No.: Shen Zilun Zi [2023] No. 029A

Name of project	Pathogenetic detection and retrospective analysis of a fatal case of Vibrio vulnificus in Shenzhen, China
Name of project leader	Xiaolu Shi
Original review approval number	——
Application Reception Number	QS2023040068
Review of conclusions	Approval
Review of comments	The sample size of this study was 2.<br>All samples in this study were isolates from hospitals after clinical diagnostic tests were done, and no original information was involved, while the information data used were anonymised, with no subsequent retrospective analyses, and did not involve the patients themselves.<br>The study is a public interest exploration of scientific knowledge and does not involve treatment or intervention, nor does it involve genetic modification or hereditary issues, and the chance of additional harm to the subjects is low.<br>Exemption from signing informed consent.<br>Consent to conduct the study.
注意事项	1. If serious adverse events occur, they should be reported to the Ethics Committee in a timely manner.<br>2. If any modification of human biomedical research trial protocol, informed consent, or change of principal investigator should be promptly notified to the Ethics Committee, re-examined, and implemented after approval.<br>3. This approval is valid for one year. Please apply for follow-up review one month before the expiration of the approval.
Tracking of reviewers	Jin Zhao<br>Jianping Liu — Tel — 13823311336<br>13823132646
Signature of the Chairperson	[illegible]<br>ETHICS COMMITTEE OF SHENZHEN CENTER FOR DISEASE CONTROL AND PREVENTION 深圳市疾病预防控制中心伦理委员会<br>盖章<br>2023年 7月 6日

Remarks: The conclusions of the review include four scenarios: approval, re-examination after revision and disapproval. Principle of numbering the approval number: the first four digits represent the year; in the middle is the acceptance sequence number; the letter A at the end represents the initial ethical review of the project, B represents the follow-up review, and C represents the revised review of the research protocol.

深圳市疾病预防控制中心伦理委员会
伦理审查批件

批件号：深疾伦字〔2023〕第 029A 号

项目名称	中国深圳 1 例创伤弧菌死亡病例的病原学检测及溯源分析		
项目负责人	石晓路		
原审查批件号	——		
申请受理号	QS2023040068		
审查结论	批准		
审查意见	本研究样本量为 2 个。<br>本研究所有样本为医院作完临床诊断检测后的分离物，不涉及原始信息，同时所使用的信息数据均经匿名化处理，后续不需要溯源分析，不涉及患者本身。<br>该研究属于公益性科学知识探索，不涉及治疗和干预，也不涉及基因改造和遗传问题，对受试者产生额外的伤害的机会很低。<br>免予签署知情同意。<br>同意开展研究。		
注意事项	1、如发生严重不良事件，应及时报告伦理委员会；<br>2、如人体生物医学研究试验方案、知情同意书的任何修改、主要研究者变更应及时通知伦理委员会，重新审查，获得批准后执行。<br>3、本批件有效期一年。请在批件到期前一个月提出跟踪审查申请。		
跟踪审查委员	赵锦<br>刘建平	联系电话	13823311336<br>13823132646
主任委员签字	[signature] 盖章<br>2023 年 7 月 6 日<br>深圳市疾病预防控制中心伦理委员会 ETHICS COMMITTEE OF SHENZHEN CENTER FOR DISEASE CONTROL AND PREVENTION		

备注：审查结论包括批准、修改后批准、修改后重审和不批准等四种情形。批件号编号原则：前四位代表年份；中间是受理序列号；后面字母 A 代表项目初次伦理审查，B 代表跟踪审查，C 代表研究方案修订审查。
